# Exploring the cross-cultural communication challenges of foreign students in China: the mediating effects of social media interaction and psychological resilience

**DOI:** 10.3389/fpsyg.2025.1560298

**Published:** 2025-05-09

**Authors:** Min Xu, Aspalila bt. Shapii

**Affiliations:** ^1^School of Education, Universiti Utara Malaysia, Sintok, Malaysia; ^2^College of Chemistry and Chemical Engineering, Xianyang Normal University, Xianyang, China

**Keywords:** foreign students in China, Intercultural Communicative Competence, Self-Determination Theory, social media interaction, psychological resilience, autonomy, competence, relatedness

## Abstract

With the development of international education, an increasing number of foreign students are choosing to study Chinese in China. However, due to cultural differences and adaptation challenges, enhancing these students’ Intercultural Communicative Competence (ICC) has become a pressing issue. Based on Self-Determination Theory (SDT), this study aims to explore how autonomy, competence, and relatedness influence the ICC of foreign students studying Chinese in China through the mediating effects of social media interaction and psychological resilience. A survey was conducted, collecting data from 500 valid samples of foreign students from various universities in China, encompassing diverse nationalities and cultural backgrounds. Structural equation modeling was employed to analyze the data. The findings reveal that autonomy, competence, and relatedness significantly and positively influence social media interaction and psychological resilience, which in turn enhance ICC. Competence emerged as the most influential factor, while psychological resilience played a critical role in cross-cultural adaptation. The results suggest that fostering autonomy, competence, and relatedness effectively enhances students’ ICC. This study provides theoretical support for the field of intercultural education and recommends that educational institutions leverage social media platforms and strategies to cultivate psychological resilience, helping students better adapt to multicultural environments.

## Introduction

The global significance of the Chinese language has been steadily rising, not only because of its prominent role in the global economy but also due to its critical importance in culture, diplomacy, and cross-cultural communication. With the advancement of the “Belt and Road Initiative, “Chinese has become an essential component of language planning in many participating countries, significantly promoting its international dissemination (Gao, 2020). Notably, the Chinese Government Scholarship has brought an increasing number of students from BRI countries to China, where Chinese language programs are often paired with cultural immersion experiences such as festival celebrations and traditional arts workshops (Shih and Cao, 2022). Through collaboration with global educational institutions, the Chinese government has been actively promoting the Chinese language, which not only enhances China’s cultural soft power but also provides global learners with a linguistic tool for engaging in international affairs (Yalun, 2019). Furthermore, the strategy of promoting Chinese is closely aligned with China’s goal of increasing its global cultural influence. Particularly in the fields of culture and diplomacy, Chinese has become a vital bridge for communication (Ding and Saunders, 2006). In Chinese universities, foreign students often rely on peer support, mentorship, and campus communities to adapt culturally, making language acquisition a lived social experience (Zheng and Ishii, 2023). Beyond being a tool for cultural exchange, the Chinese language also serves as a convergence point for different ideologies and identities in the process of globalization (Sharma, 2018).

Despite the growing international influence of the Chinese language, foreign students studying Chinese in China face numerous challenges, including language acquisition, cultural adaptation, and cross-cultural communication issues. The tonal system and complex grammatical structures of Chinese pose significant challenges for students accustomed to non-tonal languages (Gong et al., 2018). Additionally, the rules for writing Chinese characters and the differences in grammatical structures compared to many Western languages make memorizing Chinese characters particularly difficult (Gong et al., 2020). Cross-cultural communication issues, such as differences in linguistic expression and cultural backgrounds, often lead to misunderstandings and conflicts (Xu C. L., 2022). Cultural shock is especially pronounced when students’ preconceived notions of a culture differ from the reality they encounter (Xu C. L., 2022). Moreover, conflicts in verbal and non-verbal communication further increase the challenges of adaptation (Li X., 2015).

ICC is crucial in foreign language learning, as it encompasses not only language proficiency but also understanding and respecting different cultures (Byram et al., 2013). According to the ICC model proposed by Byram et al., foreign language learners must possess intercultural knowledge, skills, attitudes, and critical cultural awareness to achieve successful cross-cultural communication. Studies have shown that learners with higher levels of ICC can more effectively navigate cultural differences and demonstrate stronger adaptability in multicultural environments (Fantini, 2009). The development of ICC is particularly important for students learning Chinese in China, as it not only helps address communication barriers arising from cultural and linguistic differences but also enhances their language proficiency (DeWitt et al., 2022; Luo et al., 2021; Liang et al., 2022).

Although the importance of Intercultural Communicative Competence (ICC) has received increasing attention, there remains a significant gap in the literature regarding the underlying mechanisms that influence ICC—particularly in the context of learning Chinese as a foreign language. Firstly, while social media has been shown to play a positive role in foreign language learning, most existing studies focus on general language learners. There is a lack of in-depth exploration into how foreign students studying Chinese in China enhance their ICC through social media interaction (Nguyen et al., 2015; Simonsen, 2008). Secondly, although psychological resilience has been identified as a key psychological resource that buffers and promotes intercultural adaptation (Nomnian, 2022), its mediating role between social media interaction and ICC has yet to be systematically verified.

Moreover, few studies have adopted Self-Determination Theory (SDT) as a comprehensive theoretical framework to explain how autonomy, competence, and relatedness influence social media interaction and psychological resilience, and subsequently contribute to the development of ICC. SDT provides a coherent theoretical pathway to understand how fulfilling basic psychological needs can stimulate learning motivation and promote intercultural communication (Ryan and Deci, 2000). However, its application in the context of learning Chinese as a foreign language remains limited. Therefore, this study integrates SDT with the mediating variables of social media interaction and psychological resilience to address the above-mentioned research gaps and explore the psychological and behavioral mechanisms influencing ICC. This approach not only deepens academic understanding of theories in intercultural education but also offers practical guidance for educational institutions in designing effective support strategies to enhance foreign students’ language learning and cultural adaptation.

## Theoretical background

### Self-Determination Theory (SDT)

Self-Determination Theory (SDT) is a theoretical framework focused on human motivation, personal development, and psychological wellbeing. It emphasizes that intrinsic motivation is derived from the fulfillment of basic psychological needs, rather than external rewards or punishments (Ryan and Deci, 2000). These basic psychological needs include:

**Autonomy**: The ability of individuals to make choices and have control over their actions.

**Competence**: The capacity to manage one’s environment and successfully address challenges.

**Relatedness**: The need to establish meaningful relationships with others and feel accepted and connected.

SDT posits that satisfying these needs can stimulate intrinsic motivation, enabling individuals to achieve optimal performance and psychological health (Deci and Ryan, 2008; Deci and Ryan, 2013).

In the educational domain, SDT has been widely applied to enhance students’ learning motivation and outcomes. Research shows that supporting students’ autonomy, competence, and relatedness leads to improved learning efficiency and self-confidence (Deci et al., 1991). For cross-cultural learners, the core principles of SDT provide a solid theoretical foundation for understanding their language learning motivation and the enhancement of their Intercultural Communicative Competence (ICC).

In foreign language learning, improving ICC is a key objective. ICC encompasses not only the mastery of language skills but also understanding, respecting, and adapting to different cultures (Byram et al., 2013). SDT emphasizes that meeting learners’ basic psychological needs effectively stimulates their intrinsic motivation, thereby enhancing ICC. Specifically:

Autonomy empowers learners with a sense of control and ownership over their language learning process.

Competence helps learners build confidence when addressing language challenges.

Relatedness provides emotional and psychological support through social connections (Ryan and Deci, 2000).

Research shows that foreign students studying Chinese in China often derive their intrinsic motivation from an interest in Chinese culture and a desire for personal achievement (Wen, 1997). This intrinsic drive not only fosters deeper engagement and focus during the learning process but also strengthens their persistence and positivity when facing learning difficulties. Further studies reveal that when learners show interest in the target culture and experience a sense of accomplishment during their studies, their motivation and ICC levels significantly improve (Ruan et al., 2015).

Additionally, SDT emphasizes that the activation of intrinsic motivation also depends on a supportive learning environment. For example, classroom designs and social interactions that encourage students to engage in cross-cultural exchange activities can help them better navigate the challenges of intercultural communication (Deci et al., 2017; Tan et al., 2021). Such a supportive environment is particularly crucial for learners in cross-cultural settings, as they must not only overcome language barriers but also adapt to the social norms and communication patterns of the target culture. SDT provides an effective framework to explain why fulfilling learners’ psychological needs is instrumental in enhancing their ICC (Joe et al., 2017).

Previous studies have applied SDT to examine international students’ adjustment and learning motivation in cross-cultural contexts. For instance, Frielink et al. (2018) demonstrated that autonomy support enhances psychological wellbeing and adaptive behavior in students with special needs.

Joe et al. (2017) showed that autonomy, competence, and relatedness significantly contribute to second-language learners’ willingness to communicate and classroom engagement. Additionally, Tan et al. (2021) found that perceived autonomy-supportive and culturally responsive environments positively influence international students’ participation and intercultural interaction. While these studies confirm the relevance of SDT in educational and intercultural settings, few have explored its integration with Intercultural Communicative Competence (ICC) in the specific context of foreign students learning Chinese in China. This study extends prior work by incorporating social media interaction and psychological resilience as mediating variables, offering a more nuanced understanding of how SDT constructs influence ICC development in a Chinese language learning environment.

### Autonomy

Autonomy refers to students’ ability to have freedom of choice and make decisions for themselves during the learning process. Autonomy enables students to actively participate in learning activities, which not only helps improve their learning outcomes but also enhances the persistence of their learning efforts (Akbari et al., 2015). For foreign students, autonomy is reflected in their intrinsic motivation to independently choose to engage in cross-cultural exchanges. Such intrinsic drive is key to promoting their proactive learning and adaptation to Chinese culture. Meeting this need can enhance their engagement in cross-cultural contexts (Huang, 2022).

### Competence

Competence refers to students’ perception of their ability and sense of achievement in the learning process. Studies have shown that competence has a direct positive impact on students’ learning outcomes and can enhance their motivation to learn (Akbari et al., 2015). Competence is demonstrated through students’ mastery of language skills. When foreign students perceive an improvement in their language abilities while learning Chinese, their sense of competence is fulfilled, which further stimulates their learning motivation and promotes the development of ICC (Li X., 2015).

Relatedness emphasizes the connections and relationships students establish with others during the learning process. In a cross-cultural learning environment, relatedness can help foreign students better adapt to new cultural and linguistic settings. When students feel socially supported in their learning environment, their motivation and learning outcomes are significantly enhanced (Akbari et al., 2015). Relatedness is particularly important in intercultural communication, as students need to build connections with others in a new cultural context. Strengthening this sense of belonging helps reduce the anxiety of cultural adaptation and encourages students to actively engage in cultural exchanges, thereby enhancing ICC (Tian and Lu, 2018).

### Social media interaction

Social media interaction refers to the process through which individuals or organizations communicate, share information, and build relationships via digital platforms. Social media platforms (such as WeChat) enable users to create and share content while engaging in two-way or multi-way exchanges, thereby transforming traditional communication methods (McFarland and Ployhart, 2015). These platforms provide personalized services, enhancing user experiences (Rohm et al., 2013). Social media interaction plays a crucial role in modern business and social life.

Autonomy positively influences the social media interaction of foreign students in China. According to Self-Determination Theory (SDT), students with autonomy are more proactive in learning and engaging in cross-cultural exchanges, particularly on social media platforms, thereby enhancing their cross-cultural adaptation and communication skills (Wei et al., 2022). These students use social media more frequently, not only to seek information but also to deepen their intercultural understanding and improve their language skills through more meaningful interactions (Xu X., 2022). Research indicates that social media interaction can enhance the learning motivation of autonomous students with clear learning goals, helping them adapt more quickly to Chinese culture and society (Wang and Jiang, 2023). In summary, students’ autonomy positively influences their social media interaction, which not only facilitates cross-cultural adaptation but also improves their language learning outcomes and intercultural communication skills. Therefore, this study proposes the following hypothesis:

H1: The autonomy of foreign students learning Chinese in China positively influences their social media interaction.

Competence has a positive influence on the social media interaction of foreign students in China, particularly in the areas of language learning and cross-cultural adaptation. When students feel confident and capable in language and cultural interactions, they are more likely to actively participate in social media exchanges, thereby enhancing their language skills and intercultural communication abilities (Meng et al., 2018). This confidence motivates students to use social media for language practice and cultural interaction, effectively addressing cross-cultural challenges and accelerating their adaptation to the local environment (Xu X., 2022). Moreover, the enhancement of competence not only fosters cross-cultural communication but also strengthens language learning motivation and outcomes (Cao and Meng, 2020). In summary, competence plays a crucial role in foreign students’ social media interaction. By improving their sense of competence, students can more actively and effectively engage in cross-cultural exchanges, thereby enhancing their language proficiency and cultural adaptation skills. Therefore, this study proposes the following hypothesis:

H2: The competence of foreign students learning Chinese in China positively influences their social media interaction.

Relatedness has a significant positive impact on the social media interaction of foreign students learning Chinese in China. Relatedness helps students find psychological support in a new cultural environment, encouraging them to actively engage in interactions on platforms like WeChat and Weibo, thereby fostering connections with local and other foreign students (Nseke, 2018). These platforms serve as primary avenues for students to seek support, learn about Chinese culture, and adapt to local life, contributing to enhanced cross-cultural understanding and adaptation (Zaw, 2018). Students who feel a sense of belonging are more likely to use social media for language practice, improving their language proficiency and building meaningful social connections (Xu X., 2022). In summary, a sense of belonging is a key factor in promoting active social media interaction among foreign students, aiding their language learning, cross-cultural adaptation, and social skills development. This study proposes the following hypothesis:

H3: The relatedness of foreign students learning Chinese in China positively influences their social media interaction.

### Psychological resilience

Psychological resilience refers to an individual’s ability to adapt and maintain mental wellbeing when facing stress or adversity (Fletcherd, 2013; Wu et al., 2013). It involves actively coping with environmental challenges, enabling individuals to recover quickly and maintain a positive psychological state in difficult situations, thereby reducing the risk of anxiety, depression, and other mental health issues (Shi et al., 2019). Additionally, psychological resilience helps enhance social adaptability, allowing individuals to adjust their behavior patterns more flexibly in complex environments (Sisto et al., 2019). Studies have shown that psychological resilience acts as a buffer in various contexts. For instance, when facing stress, it can effectively mitigate the negative impact of stress on mental health, facilitating quicker adaptation to new environments (Luo et al., 2024).

In cross-cultural contexts, psychological resilience is regarded as a critical factor in enhancing cross-cultural adaptation. For foreign students studying in China, psychological resilience strengthens their ability to cope with cultural differences and language barriers, enabling them to engage more actively in social interactions and significantly improving the effectiveness of intercultural communication (Üzar-Özçetin et al., 2022). Specifically, psychological resilience not only helps students manage the stress associated with cross-cultural environments but also facilitates their faster integration into the target cultural setting, improving their learning outcomes and the quality of their social interactions. This highlights that psychological resilience serves as a key psychological resource supporting students in adapting to multicultural environments.

Self-Determination Theory (SDT) emphasizes that fulfilling individuals’ basic psychological needs for autonomy, competence, and relatedness can stimulate intrinsic motivation and enhance psychological wellbeing and adaptability (Ryan and Deci, 2000). In the context of cross-cultural learning, meeting these needs plays a vital role in improving students’ psychological resilience. For instance, when students experience autonomy in the learning process, they are better equipped to handle cultural shocks and challenges in language learning, thereby strengthening their psychological resilience (Frielink et al., 2018). Specifically, autonomy support enables students to develop proactive coping strategies, enhancing their adaptability to new environments and their ability to manage stress. Based on theoretical and empirical studies, this research proposes the following hypothesis:

H4: The autonomy of foreign students learning Chinese in China positively influences their psychological resilience.

Competence positively influences the psychological resilience of foreign students studying in China. When students feel capable of completing their academic tasks, their confidence is strengthened, enabling them to better cope with challenges and stress during the learning process. Research indicates that fulfilling the need for competence significantly enhances students’ adaptability and psychological resilience (Perlman et al., 2017). Based on this, the following hypothesis is proposed:

H5: The competence of foreign students learning Chinese in China positively influences their psychological resilience.

Relatedness also positively impacts the psychological resilience of foreign students studying in China. Relatedness provides students with feelings of support when faced with a new cultural environment, enhancing their resilience in managing stress. Studies suggest that relatedness helps students establish a sense of psychological safety through social support, thereby boosting their ability to withstand pressure and improving their psychological resilience (Patrick et al., 2007). Based on this, the following hypothesis is proposed:

H6: The relatedness of foreign students learning Chinese in China positively influences their psychological resilience.

### Intercultural Communication Competence (ICC)

Intercultural Communication Competence (ICC) refers to an individual’s ability to communicate effectively and appropriately across different cultural contexts. It is a multidimensional competence that involves language knowledge, cultural understanding, and communication skills (Chen and Starosta, 2012). The core components of ICC include motivation, knowledge, and skills, which together enable individuals to accurately interpret and understand others’ intentions in multicultural environments, thereby achieving communication objectives (Arasaratnam and Doerfel, 2005). ICC is essential for understanding and collaboration in multicultural settings, as it helps to avoid misunderstandings and conflicts caused by cultural differences while facilitating cooperation in diverse contexts (Beamer, 1992).

In today’s diverse society, the importance of Intercultural Communication Competence (ICC) has become increasingly prominent, particularly in fields such as education, international trade, and diplomacy. ICC effectively facilitates cross-cultural interactions and helps individuals adapt and thrive in multicultural environments. It not only enhances individuals’ understanding of other cultures but also supports the establishment of trust and cooperative social relationships, thereby achieving effective cross-cultural communication (Ruben, 2015). This competence enables individuals to navigate cultural differences more flexibly, improving the success rate of cross-cultural communication and fostering social integration (Ilie, 2019).

Social media platforms, such as WeChat, provide foreign students with convenient channels to interact with local students and other international peers. This not only helps them adapt more quickly to Chinese cultural norms but also enhances their Intercultural Communication Competence (ICC). Research indicates that the use of social media significantly facilitates cultural adaptation and intercultural understanding, thereby improving ICC (Zhou, 2020). These platforms also offer students opportunities to practice intercultural skills, manage cultural conflicts, and support their success in both daily life and academic settings (Yu and Guo, 2021). Specifically, for foreign students learning Chinese in China, engaging in language exchanges and cultural interactions via social media has been shown to improve their Chinese language proficiency and cultural understanding, thereby enhancing their ICC (Monika et al., 2020; Nseke, 2018).

Specifically, social media platforms help students establish supportive social networks, enabling them to adapt more quickly to the target culture and respond flexibly to cultural differences and potential conflicts (Yu and Guo, 2021). Frequent social media interactions not only improve students’ language skills but also deepen their understanding of Chinese culture, further enhancing their performance in intercultural contexts (Na). As a result, social media has become a crucial tool for promoting the improvement of ICC among foreign students. Based on the above literature, this study proposes the following hypothesis:

H7: Social media interaction positively influences the Intercultural Communication Competence (ICC) of foreign students learning Chinese in China.

The psychological resilience of foreign students enhances their stability and adaptability in cross-cultural situations, enabling them to exhibit greater flexibility and proactiveness when facing cultural differences (Chu and Zhu, 2023). Psychological resilience acts as a buffer against the stress of cross-cultural adaptation, helping students better adjust to and understand the local culture. Students with high resilience are more confident in participating in intercultural interactions, thereby improving their language and cultural communication skills. Research indicates that psychological traits such as emotional resilience contribute to better communication performance in intercultural contexts (Taguchi et al., 2016). The psychological resilience of foreign students allows them to manage emotions effectively when encountering cultural conflicts, facilitating smoother intercultural communication. This resilience not only boosts their confidence in communication but also strengthens their tolerance and understanding of cultural differences (Sun and Buys, 2013).

Based on the above, this study proposes the following hypothesis:

H8: The psychological resilience of foreign students learning Chinese in China positively influences their Intercultural Communication Competence (ICC).

Autonomy helps students exhibit proactivity and control in cross-cultural interactions, thereby enhancing their intercultural understanding and adaptability (Lee, 2011). When students possess higher autonomy, their engagement on social media increases, and their ability to independently choose interaction content and methods further promotes their communication skills and cultural adaptation (Ruan and Medwell, 2020). Social media provides students with diverse opportunities for cross-cultural exchanges. Autonomous students can leverage these interactions to practice and develop their ICC, improving their tolerance and understanding of cultural differences (Taguchi et al., 2016). Based on this, the study proposes the following hypothesis:

H9: The autonomy of foreign students learning Chinese in China positively influences their Intercultural Communication Competence (ICC) through social media interaction.

Competence enhances students’ confidence, making them more proactive and effective in cross-cultural interactions on social media (Sheu, 2018). When students perceive their own competence, their ability to actively address challenges and adapt to cultural differences on social media is strengthened, which translates into higher levels of ICC. Social media provides a platform for competent students to practice and expand their cultural understanding and communication skills, further supporting the development of their ICC (Xu X., 2022). Through interactions with locals or other foreign students, they can improve their language proficiency and reinforce their cross-cultural adaptability (Wang et al., 2012). Based on this, the study proposes the following hypothesis:

H10: The competence of foreign students learning Chinese in China positively influences their Intercultural Communication Competence (ICC) through social media interaction.

Relatedness alleviates feelings of loneliness during the cultural adaptation process, making students more willing to interact with locals and peers through social media, thereby building supportive social networks (Li X., 2015). This relatedness encourages students to actively participate in cultural exchange activities and integrate more deeply into the target culture through social media platforms (Teng and Bui, 2020). Through these interactions, students not only enhance their understanding of the target culture but also improve their adaptability to cultural differences and intercultural skills (Ruan and Medwell, 2020). A sense of belonging serves as a bridge in intercultural communication, enabling students to enhance their ICC through social media interaction. Based on this, the study proposes the following hypothesis:

H11: The relatedness of foreign students learning Chinese in China positively influences their Intercultural Communication Competence (ICC) through social media interaction.

Autonomy provides students with a sense of control and freedom in their actions during the process of cultural adaptation, enhancing their ability to cope with cultural shocks (Ryan and Deci, 2000). Through psychological resilience, autonomy helps students handle challenges in cross-cultural contexts more flexibly, improving their adaptability in new cultural environments and ultimately strengthening their ICC (Ruan and Medwell, 2020). Based on this, the study proposes the following hypothesis:

H12: The autonomy of foreign students learning Chinese in China positively influences their Intercultural Communication Competence (ICC) through psychological resilience.

Competence enhances students’ confidence in facing cultural differences and language challenges, equipping them with the ability to handle difficulties in a new environment (Taguchi et al., 2016). Psychological resilience acts as a buffering mechanism, helping students maintain a stable mindset amid stress and conflict, thereby improving their cultural adaptation and communication outcomes (Sung and Guo, 2020). The combination of competence and psychological resilience provides students with greater flexibility and confidence, enabling them to demonstrate higher ICC in cross-cultural interactions (Li X., 2015). Based on this, the study proposes the following hypothesis:

H13: The competence of foreign students learning Chinese in China positively influences their Intercultural Communication Competence (ICC) through psychological resilience.

Relatedness helps students reduce feelings of loneliness during the cultural adaptation process, boosting their resilience and confidence in integrating into the culture (Teng and Bui, 2020). Through psychological resilience, a sense of belonging enables students to maintain a positive mindset when facing cultural differences, effectively addressing cross-cultural challenges and enhancing their ICC (Li X., 2015). The interaction between a sense of belonging and psychological resilience allows students to handle cultural conflicts more flexibly and achieve successful cultural adaptation in cross-cultural contexts (Taguchi et al., 2016). Based on this, the study proposes the following hypothesis:

H14: The relatedness of foreign students learning Chinese in China positively influences their Intercultural Communication Competence (ICC) through psychological resilience.

### Research participants and data collection

This study targets foreign students studying Chinese at universities in China. Since 2000, China has actively developed itself as an international education hub, attracting a significant number of foreign students to study in the country (Botha, 2016). According to statistics, in 2018, a total of 492,185 foreign students from 196 countries and regions were enrolled in 1,004 higher education institutions across 31 provinces, autonomous regions, and municipalities in China. This represented an increase of 3,013 students, or a growth rate of 0.62%, compared to 2017 (Ministry_of_Education_of_PRC, 2019).

The study utilized an online questionnaire distributed through foreign student groups on social media platforms. Data collection was conducted from September 30, 2024, to October 31, 2024. Using the Sample Size Calculator provided by Creative Research Systems, with a confidence level of 95% and a margin of error of 5%, a sample size of 384 responses was calculated based on an estimated population of 500,000. This study collected 486 valid responses, meeting the requirement for effective sample size.

#### Measurement tools

The questionnaire used in this study is divided into two parts. Although a formal pilot test was not conducted, the questionnaire was developed using well-established scales from prior validated studies and was carefully reviewed to ensure clarity and contextual relevance for international students in China.

Demographic variables: This section includes questions about gender, age, region, Chinese language proficiency, and duration of stay in China. Research constructs: This section employs Likert-scale measures to assess the key constructs of the study. The constructs include autonomy, competence, sense of belonging, social media interaction, psychological resilience, and Intercultural Communication Competence (ICC).

In measuring autonomy, competence, and relatedness, this study references the work of Nikou and Economides (2017). Their research, which integrates Self-Determination Theory (SDT) and the Technology Acceptance Model (TAM), focuses on examining the acceptance and motivational factors of Mobile-Based Assessment (MBA). SDT emphasizes that autonomy, competence, and relatedness are the three essential psychological needs that promote intrinsic motivation. Thus, their study provides a theoretical foundation for understanding how these three constructs influence students’ learning motivation and behavior during the learning process. The context of their research, involving students using technological tools for learning, shares similarities with the situation of students learning Chinese in this study. Specifically, the autonomy, competence, and sense of belonging experienced by students during the learning process have profound impacts on their learning outcomes and acceptance of technology. This makes adopting their constructs and scales a rational choice for this study. Based on the context of this research, the items related to autonomy, competence, and relatedness were adapted to assess the constructs in the context of international students studying Chinese in China. A total of 12 items were developed for these constructs.

For the social media interaction construct, this study references the scale proposed by Sahin (2018), which has demonstrated good reliability and validity in measuring students’ attitudes, addiction levels, and behaviors related to social media usage. This scale includes multiple items capturing various dimensions of social media use, specifically tailored for students. Six items from Sahin’s scale were adapted and rewritten to suit the context of this study. For the psychological resilience construct, this study employs items based on the “Connor-Davidson Resilience Scale (CD-RISC)” developed by Connor and Davidson (2003). This widely cited and classic tool for measuring resilience has been validated across various contexts and is known for its high reliability and validity. The scale emphasizes the importance of resilience in dealing with stress, challenges, and adversity, with items related to coping ability, optimism, and a sense of control. A total of six items were modified from the CD-RISC to reflect the application of resilience in different scenarios, including coping with challenges, managing stress, and maintaining optimism. These items are designed to capture individuals’ strategies and capabilities in facing difficulties, making them suitable for studying the psychological resilience of foreign students in cross-cultural adaptation.

The Intercultural Communication Competence (ICC) items in this study are designed based on four operationally defined dimensions: awareness, attitude, skills, and knowledge. Each dimension’s items aim to assess individuals’ abilities and performance in intercultural communication. Awareness items evaluate individuals’ recognition and acceptance of cultural differences. Attitude items focus on individuals’ willingness to interact with locals and learn about the local culture. Skills items measure individuals’ flexibility and adaptive strategies in intercultural exchanges. Knowledge items assess individuals’ understanding of cultural differences and their ability to learn the local culture and language. These items are adapted from the intercultural competence questionnaire developed by Shi (2016). Shi’s study provides valid data support and primarily explores the dynamic relationship between intercultural competence (ICC) and language learning among Chinese immigrant students. Using a case study approach, the research focuses on Chinese immigrant students, aiming to understand how they adapt to new cultural environments and how the development of ICC influences their language learning.

To measure the key variables in this study, a structured questionnaire was developed using validated scales, adapted slightly to fit the context of international students learning Chinese in China. The following table summarizes each variable, the number of items included, and two sample questions to illustrate the measurement approach as Table 1 shown.

**Table 1:** Variables and sample items.

Variable	Number of items	Sample items
Autonomy (AUT)	4 items	1. In the process of learning Chinese, I feel there is room for choice and freedom. 2. I have many opportunities to decide my own learning methods and pace while learning Chinese.
Competence (COMP)	4 items	1. I believe I am doing well in learning Chinese. 2. After spending some time learning Chinese, I feel I have become more capable.
Relatedness (REL)	4 items	1. I feel closely connected to my classmates. 2. I have opportunities to build close relationships with other classmates.
Social Media Interaction (SMI)	6 items	1. I often communicate with classmates through social media. 2. I feel that social media can help me integrate more quickly into the Chinese cultural environment.
Resilience (RES)	6 items	1. Even when I encounter difficulties, I remain optimistic. 2. When I face challenges, I am able to quickly find ways to cope.
ICC – Awareness (ICC_AW)	4 items	1. I am able to recognize differences across languages and cultures. 2. I am aware of how members of the local country perceive me.
ICC – Attitude (ICC_AT)	4 items	1. I am willing to interact with people from the local country. 2. I am willing to try using language and behavior that locals consider “appropriate” for communication.
ICC – Skill (ICC_SK)	5 items	1. My behavior shows flexibility when interacting with people from the host culture. 2. I develop strategies to learn the local culture and language.
ICC – Knowledge (ICC_KN)	5 items	1. I can understand the differences between the local culture and my own culture. 2. I am aware of the basic norms and taboos of the local culture.

### Data analysis

**Figure 1:**
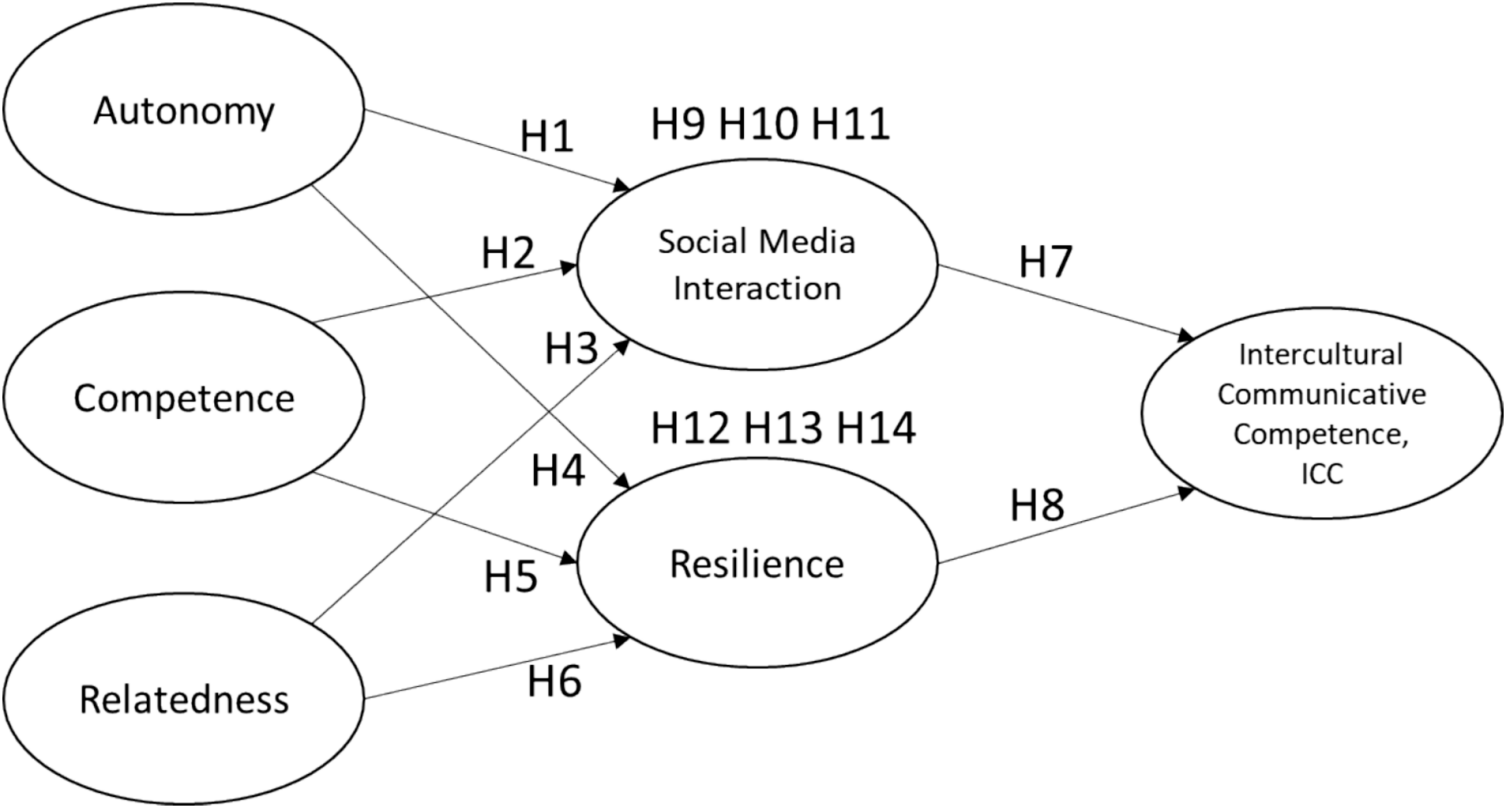
Research framework.

This study will use SPSS 24.0 and Smart PLS 4.0 as statistical analysis tools, conducting data processing and verification in three stages: descriptive statistical analysis, measurement model analysis, and structural model analysis. First, in the descriptive statistical analysis stage, SPSS is used for sample description, calculating the sample characteristics distribution and central tendencies. Next, in the measurement model analysis stage, Smart PLS 4.0 is employed to perform Confirmatory Factor Analysis (CFA) to assess the reliability and validity of each construct, including composite reliability (CR), Cronbach’s *α*, convergent validity (Average Variance Extracted, AVE), and discriminant validity (Fornell-Larcker criterion). Finally, in the structural model analysis stage, the model’s fit with the sample data and research hypotheses are tested. Path analysis is conducted through PLS to evaluate the standardized coefficients (*β* values) and their significance (*p*-values). The bootstrapping method is used to examine the significance of mediation effects (*p*-values). The explanatory power of the model on the dependent variables is reported using the *R*^2^ values. The statistical methods used in this study aim to ensure the rigor and interpretability of the model and results (Figure 1 and Table 2).

**Table 2:** Analysis of demographic data.

Category	Group	Frequency	Percentage (%)
Gender	Male	315	64.8
	Female	171	35.2
Age	Under 20	175	36.0
	20–25 age	213	43.8
	Above 25	98	20.2
Region	Asia	233	47.9
	Europe	15	3.1
	Americas	33	6.8
	Africa	205	42.2
Chinese proficiency level	Beginner	202	41.6
	Intermediate	247	50.8
	Advanced	37	7.6
Time spent in China	Less than 1 year	188	38.7
	1–3 years	242	49.8
	More than 3 years	56	11.5

## Research results

### Analysis of demographic data

The characteristics of the sample show that the majority of participants are male, with 315 respondents accounting for 64.8% of the total sample. The age group is primarily concentrated between 20 and 25 years, with 213 respondents making up 43.8%. Regarding regions, most participants are from Asia, with 233 respondents, representing 47.9%. In terms of Chinese proficiency, the majority of respondents are at the intermediate level, with 247 participants, accounting for 50.8% as shown in Table 3.

**Table 3:** Convergent validity analysis.

Construct	Item	Factor loadings	Cronbach’s alpha	Composite reliability	Average variance extracted (AVE)
Autonomy	AUT1	0.936	0.894	0.927	0.760
	AUT2	0.896			
	AUT3	0.805			
	AUT4	0.844			
Competence	COMP1	0.872	0.888	0.922	0.748
	COMP2	0.831			
	COMP3	0.895			
	COMP4	0.861			
Intercultural Communicative Competence attitude	ICC_AT1	0.876	0.886	0.922	0.746
	ICC_AT2	0.805			
	ICC_AT3	0.896			
	ICC_AT4	0.875			
Intercultural Communicative Competence awareness	ICC_AW1	0.843	0.838	0.892	0.674
	ICC_AW2	0.811			
	ICC_AW3	0.835			
	ICC_AW4	0.793			
intercultural communicative competence knowledge	ICC_KN1	0.828	0.889	0.918	0.693
	ICC_KN2	0.782			
	ICC_KN3	0.861			
	ICC_KN4	0.856			
	ICC_KN5	0.832			
Intercultural Communicative Competence skill	ICC_SK1	0.847	0.870	0.906	0.659
	ICC_SK2	0.760			
	ICC_SK3	0.796			
	ICC_SK4	0.827			
	ICC_SK5	0.827			
Relatedness	REL1	0.862	0.926	0.948	0.819
	REL2	0.941			
	REL3	0.914			
	REL4	0.900			
Resilience	RES1	0.845	0.911	0.931	0.692
	RES2	0.813			
	RES3	0.856			
	RES4	0.873			
	RES5	0.819			
	RES6	0.783			
Social media interaction	SMI1	0.737	0.886	0.914	0.638
	SMI2	0.791			
	SMI3	0.839			
	SMI4	0.836			
	SMI5	0.785			
	SMI6	0.801			
Intercultural Communicative Competence	AT	0.885	0.930	0.950	0.827
	AW	0.903			
	KN	0.913			
	SK	0.936			

### Convergent validity

According to the evaluation criteria for validity as proposed by Fornell and Larcker (1981) and Nunnally (1978), the standards for assessing the measurement model include the following: factor loadings must exceed 0.7, composite reliability (CR) must be greater than 0.7, the average variance extracted (AVE) must be greater than 0.5, and Cronbach’s *α* should be greater than 0.7. The statistical analysis reveals the following results for this study:

Factor loadings range between 0.760 and 0.941, all exceeding 0.7. Composite reliability for all constructs ranges from 0.892 to 0.950, demonstrating CR values greater than 0.7.

Average variance extracted (AVE) values range from 0.638 to 0.827, indicating all AVE values exceed 0.5.

Cronbach’s *α* values for the constructs range from 0.838 to 0.930, confirming that all values exceed 0.7.

These results indicate that the measurement model exhibits good convergent validity. Table 3 provides a detailed summary of the convergent validity analysis.

### Discriminant validity

This study evaluates the discriminant validity of reflective constructs using the method of Average Variance Extracted (AVE). According to the criteria of Fornell and Larcker (1981), discriminant validity is established when the square root of each construct’s AVE is greater than its correlation coefficients with other constructs. The analysis results indicate that, for most constructs in this study, the square root of AVE values exceeds the squared correlations between constructs, meeting the requirements for discriminant validity. This demonstrates that sufficient distinctions exist between reflective constructs, and each construct effectively captures a unique concept. Overall, the study achieves good discriminant validity, as shown in Table 4.

**Table 4:** Discriminant validity analysis.

Construct	Autonomy	Competence	Intercultural Communicative Competence	Relatedness	Resilience	Social media interaction
Autonomy	**0.872**					
Competence	0.667	**0.865**				
Intercultural communicative competence	0.581	0.666	**0.909**			
Relatedness	0.602	0.616	0.529	**0.905**		
Resilience	0.564	0.575	0.721	0.524	**0.832**	
Social media interaction			0.665	0.628	0.574	**0.799**

Bold diagonal values represent the square root of AVE, used to assess discriminant validity based on the Fornell-Larcker criterion.

### Model fit

The Goodness of Fit (GOF) index is calculated as *GOF* = $\sqrt{AVE}\times \sqrt{R^{2}}$ , which serves as an overall measure of the model’s fit. According to Vinzi et al. (2010), a GOF value of 0.1 indicates weak fit, 0.25 indicates moderate fit, and 0.36 indicates strong fit. The GOF value for this study is 0.613, indicating a strong model fit. This result demonstrates that the measurement model exhibits a high level of overall fit.


$$GOF=\sqrt{AVE}\times \sqrt{R^{2}}=\sqrt{0.726\times 0.518}=0.613$$

### Path analysis

The results of this study indicate that the effects of all paths reach a significant level, demonstrating that the independent variables have a significant predictive and influential power on the related endogenous variables.

#### Impact of autonomy

The path coefficient of Autonomy on Social Media Interaction is 0.189 (*t*-value = 2.944, *p* = 0.003 < 0.05), indicating a significant positive impact of Autonomy on Social Media Interaction.

The path coefficient of Autonomy on Resilience is 0.258 (*t*-value = 3.427, *p* = 0.001 < 0.05), demonstrating a significant positive impact of Autonomy on Resilience.

The influence of Autonomy on Resilience is stronger than its influence on Social Media Interaction.

#### Impact of competence

The path coefficient of Competence on Social Media Interaction is 0.348 (*t*-value = 4.920, *p* = 0.000 < 0.05), indicating a significant positive impact of Competence on Social Media Interaction.

The path coefficient of Competence on Resilience is 0.284 (*t*-value = 3.800, *p* = 0.000 < 0.05), demonstrating a significant positive impact of Competence on Resilience.

The influence of Competence on Social Media Interaction is stronger than its influence on Resilience.

#### Impact of relatedness

The path coefficient of Relatedness on Social Media Interaction is 0.300 (*t*-value = 4.867, *p* = 0.000 < 0.05), indicating a significant and relatively strong positive impact of Relatedness on Social Media Interaction.

The path coefficient of Relatedness on Resilience is 0.193 (*t*-value = 3.059, *p* = 0.002 < 0.05), demonstrating a significant positive impact of Relatedness on Resilience.

The influence of Relatedness on Social Media Interaction is stronger than its influence on Resilience.

#### Impact of social media interaction and resilience

The path coefficient of Social Media Interaction on Intercultural Communicative Competence (ICC) is 0.374 (*t*-value = 7.597, *p* = 0.000 < 0.05), indicating a significant positive impact of Social Media Interaction on ICC.

The path coefficient of Resilience on Intercultural Communicative Competence (ICC) is 0.506 (*t*-value = 10.254, *p* = 0.000 < 0.05), demonstrating a significant and strong positive impact of Resilience on ICC.

The research results indicate that Autonomy, Competence, and Relatedness have significant positive impacts on both Resilience and Social Media Interaction. Additionally, Resilience and Social Media Interaction exhibit significant and strong positive effects on Intercultural Communicative Competence (ICC). These findings support the hypotheses in the research model and confirm the explanatory power of the independent variables and mediating variables on the endogenous variable. Furthermore, the results suggest that fostering Autonomy, Competence, and Relatedness can indirectly enhance ICC by improving Psychological Resilience and Social Media Interaction, as shown in Table 5.

**Table 5:** Path analysis.

Path relationship	Path	Standard deviation	*t* value	*p* value
H1: Autonomy → Social Media	0.189	0.064	2.944	0.003
H2: Competence → Social Media	0.348	0.071	4.920	0.000
H3: Relatedness → Social Media	0.300	0.062	4.867	0.000
H4: Autonomy → Resilience	0.258	0.075	3.427	0.001
H5: Competence → Resilience	0.284	0.075	3.800	0.000
H6: Relatedness → Resilience	0.193	0.063	3.059	0.002
H7: Social Media Interaction → Intercultural Communicative Competence	0.374	0.049	7.597	0.000
H8: Resilience → Intercultural Communicative Competence	0.506	0.049	10.254	0.000

In practical terms, the coefficient for psychological resilience (*β* = 0.506) suggests a strong influence on ICC. This means that students with higher resilience—those who can effectively cope with stress and adapt to new environments—are more capable of navigating cultural differences and participating in intercultural communication with confidence. For instance, they may be more likely to persist in conversations despite language barriers or engage more openly in cultural exchange activities.

Similarly, social media interaction (*β* = 0.374) has a meaningful effect on ICC, indicating that students who frequently use platforms such as WeChat to communicate with peers are more exposed to authentic cultural content and social norms. These interactions help build cultural understanding, reduce misunderstandings, and improve both language proficiency and intercultural sensitivity. These findings highlight that both internal traits like resilience and external practices like social media engagement contribute substantially to students’ development of Intercultural Communicative Competence in everyday life.

**Figure 2:**
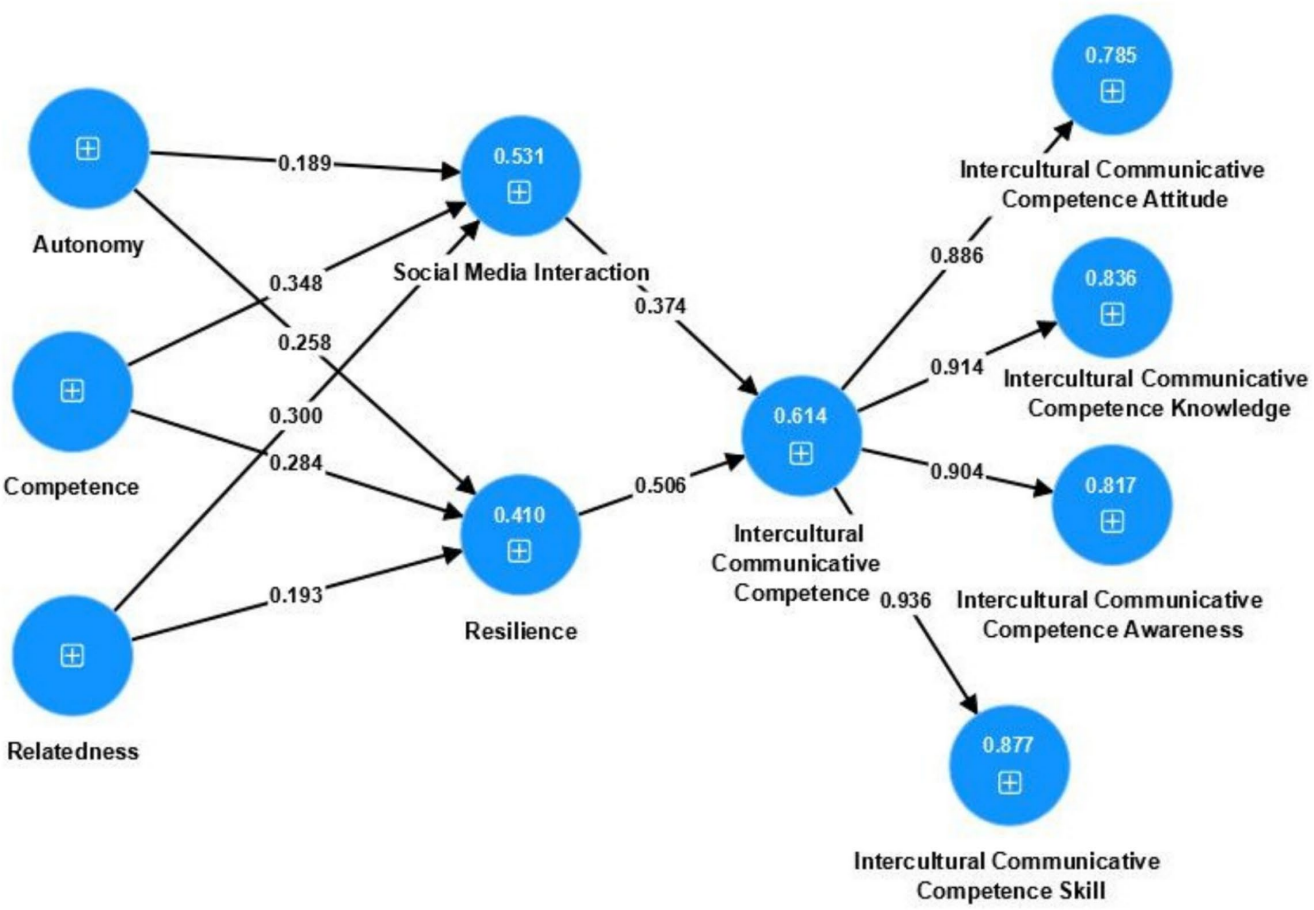
PLS-SEM statistical model diagram.

From Figure 2, the *R*^2^ values of the structural model can be observed, representing the explanatory power of the endogenous variables.

The *R*^2^ value for Social Media Interaction is 0.531, indicating that Autonomy, Competence, and Relatedness collectively explain 53.1% of its variance. This demonstrates that the model’s explanatory power for Social Media Interaction is moderate to high (typically, *R*^2^ > 0.50 is considered high explanatory power).

The *R*^2^ value for Resilience is 0.410, showing that Autonomy, Competence, and Relatedness explain 41.0% of its variance. This represents moderate explanatory power, indicating that the model has a fair predictive capacity for Resilience.

The *R*^2^ value for Intercultural Communicative Competence (ICC) is 0.614, meaning that Social Media Interaction and Resilience together explain 61.4% of its variance. This indicates high explanatory power, suggesting that these two antecedent variables have a significant impact on ICC.

These findings are illustrated in Figure 2: PLS-SEM Statistical Model Diagram.

### Mediation effects

The results of this study indicate that multiple mediation effects in the model reach significant levels, and the confidence intervals do not include 0, supporting the existence of mediation effects.

#### Social media interaction as a mediating variable

H9: Autonomy → Social Media Interaction → Intercultural Communicative Competence: The mediation effect is significant (*p* < 0.05), with a confidence interval of [0.022, 0.122] not containing 0. This demonstrates that Autonomy has an indirect impact on Intercultural Communicative Competence (ICC) through Social Media Interaction.

H10: Competence → Social Media Interaction → Intercultural Communicative Competence: The mediation effect is significant (*p* < 0.05), with a confidence interval of [0.072, 0.201] not containing 0. This indicates that Competence can indirectly enhance ICC through Social Media Interaction.

H11: Relatedness → Social Media Interaction → Intercultural Communicative Competence: The mediation effect is significant (*p* < 0.05), with a confidence interval of [0.063, 0.166] not containing 0. This confirms that Relatedness can indirectly influence ICC through Social Media Interaction.

#### Resilience as a mediating variable

H12: Autonomy → Resilience → Intercultural Communicative Competence: The mediation effect is significant (*p* < 0.05), with a confidence interval of [0.052, 0.212] not containing 0. This indicates that Autonomy can indirectly enhance Intercultural Communicative Competence (ICC) through Resilience.

H13: Competence → Resilience → Intercultural Communicative Competence: The mediation effect is significant (*p* < 0.05), with a confidence interval of [0.068, 0.228] not containing 0. This demonstrates that Competence can indirectly exert a positive impact on ICC through Resilience.

H14: Relatedness → Resilience → Intercultural Communicative Competence: The mediation effect is significant (*p* < 0.05), with a confidence interval of [0.037, 0.166] not containing 0. This confirms that Relatedness can indirectly influence ICC through Resilience.

The results of this study confirm that Social Media Interaction and Resilience play significant mediating roles in the model. Autonomy, Competence, and Relatedness not only have direct effects on Intercultural Communicative Competence (ICC) but also exert indirect effects through these two mediating variables. This finding suggests that autonomy, competence, and relatedness can further enhance individuals’ Intercultural Communication Competence by improving social media interaction and psychological resilience. The mediating effects in the model provide deeper theoretical support for this framework and highlight the importance of mediating variables, as shown in Table 6.

**Table 6:** Mediation effect analysis.

Mediation effect	Path coefficient	Standard deviation	*t* value	*p* value	2.50%	97.50%
Relatedness → Social Media Interaction → Intercultural Communicative Competence	0.112	0.027	4.242	0.000	0.063	0.166
H9: Autonomy → Social Media Interaction → Intercultural Communicative Competence	0.071	0.026	2.758	0.006	0.022	0.122
Competence → Social Media Interaction → Intercultural Communicative Competence	0.130	0.033	3.968	0.000	0.072	0.201
Relatedness → Resilience → Intercultural Communicative Competence	0.098	0.033	2.987	0.003	0.037	0.166
Autonomy → Resilience → Intercultural Communicative Competence	0.131	0.041	3.217	0.001	0.052	0.212
Competence → Resilience → Intercultural Communicative Competence	0.144	0.041	3.497	0.000	0.068	0.228

## Conclusion and discussion

### Research conclusions

#### The influence of autonomy, competence, and relatedness on social media interaction

The research findings indicate that autonomy, competence, and relatedness all have a significant positive influence on social media interaction among international students in China, though the strength of these effects varies.

Firstly, the impact of autonomy on social media interaction, while significant, is relatively weaker. This suggests that students with higher levels of autonomy are more actively engaged in social media interactions, such as seeking learning resources or engaging in cross-cultural communication with local Chinese students, thereby enhancing their language skills and cultural understanding. However, the relatively weak influence of autonomy may be due to the fact that cross-cultural interaction requires external conditions, such as language proficiency and social support, in addition to intrinsic motivation.

Secondly, competence exerts the strongest influence on social media interaction. This highlights that when students feel confident in their language and cultural interaction abilities, they are more proactive in participating in language practice and cultural exchange, effectively improving their language skills and cross-cultural adaptation. This finding aligns with related literature, such as studies by Meng et al. (2018) and Cao and Meng (2020), which emphasize that competence not only reduces barriers to cross-cultural communication but also significantly enhances students’ learning motivation and adaptability.

Finally, relatedness has a moderate impact on social media interaction. Students who feel a sense of belonging are more likely to build supportive relationships through social media, such as participating in group chats on WeChat or discussions on Weibo, further promoting language learning and cultural adaptation. This finding is consistent with studies by Nseke (2018) and Zaw (2018), which confirm that the relatedness helps students find psychological support in a new cultural environment, thereby encouraging more frequent and in-depth social interactions.

Overall, competence has the strongest impact, likely because the effectiveness of language and cultural interactions directly depends on students’ confidence and abilities. Relatedness follows, as it primarily influences students’ social motivation and psychological support. Although autonomy serves as a crucial source of intrinsic motivation, it may require additional external factors to fully exert its effect in cross-cultural interactions.

#### The relationship between autonomy, competence, and relatedness on psychological resilience

The research findings indicate that autonomy, competence, and relatedness all have significant positive effects on the psychological resilience of international students learning Chinese in China, though the strength of these effects varies.

Competence has the most significant impact on psychological resilience, suggesting that when students feel capable of completing learning tasks, their confidence is enhanced, enabling them to more effectively cope with academic and cross-cultural challenges. This aligns with the findings of Perlman et al. (2017). Autonomy follows, indicating that when students feel autonomous, they can devise more flexible coping strategies, thereby improving their ability to handle stress, supporting the theory proposed by Ryan and Deci (2000). However, autonomy may need to be paired with competence to be more effectively transformed into actionable motivation, leading to a slightly lower impact compared to competence.

The influence of relatedness is relatively weaker but still significant, demonstrating that relatedness can enhance students’ resilience through social support and a sense of psychological safety, consistent with the findings of Patrick et al. (2007).

Overall, competence provides direct support in terms of ability and confidence, making it the core factor in enhancing psychological resilience. Autonomy offers intrinsic motivation, helping students proactively address challenges, while relatedness plays a supplementary role by fostering psychological support.

#### The influence of social media interaction and psychological resilience on Intercultural Communication Competence (ICC)

The research findings indicate that both social media interaction and psychological resilience have significant positive effects on the Intercultural Communication Competence (ICC) of international students learning Chinese in China, though the strength of these effects differs.

Psychological resilience has the most significant impact on ICC, demonstrating that resilience helps students remain stable and adaptable when facing cultural differences, enhancing their confidence and ability to engage in intercultural interactions. Students with higher psychological resilience can better manage emotions, facilitating smooth resolution of cultural conflicts and fostering inclusivity and cultural understanding. This aligns with the findings of Chu and Zhu (2023) and Taguchi et al. (2016), which emphasize the central role of resilience in cross-cultural adaptation.

In comparison, social media interaction also significantly impacts ICC but to a lesser extent. Through platforms such as WeChat, students can engage in frequent language practice and cultural exchange, improving language skills, cultural understanding, and building supportive social networks. This finding is consistent with studies by Zhou (2020) and Yu and Guo (2021).

Psychological resilience, as an intrinsic psychological trait, has a more enduring and profound impact on ICC. In contrast, social media interaction, as an external factor, may depend on the frequency and depth of students’ engagement for its effectiveness.

### Mediation effects

The research findings indicate that autonomy, competence, and relatedness significantly influence the Intercultural Communication Competence (ICC) of international students learning Chinese in China, and they exert indirect effects through social media interaction and psychological resilience. The direct and indirect effects of autonomy, competence, and relatedness on ICC vary in strength. Additionally, the mediating roles of social media interaction and psychological resilience differ in their impact.

#### Mediating effect of social media interaction

The study reveals that social media interaction plays an important mediating role in the relationship between autonomy, competence, relatedness, and ICC. Autonomy exerts a significant but relatively weak indirect effect on ICC through social media interaction. The mediating effect of competence is more pronounced, indicating that competence encourages students to confidently use social media for intercultural interactions, thereby significantly enhancing ICC. Relatedness also shows a mediating effect, demonstrating that it helps students build supportive social networks and improve cultural understanding through social media. These findings are consistent with literature such as Lee (2011) and Xu C. L. (2022), which emphasize that social media provides an essential platform for intercultural communication among students.

#### Mediating effect of psychological resilience

Psychological resilience also plays a significant mediating role in the relationship between autonomy, competence, relatedness, and ICC. The indirect effect of autonomy on ICC through psychological resilience shows that autonomy helps students adapt flexibly to stress in cross-cultural contexts, thereby improving their communication abilities. Competence demonstrates the strongest indirect effect among all pathways, indicating that students with higher competence can engage more effectively in intercultural interactions with the support of psychological resilience. The mediating effect of relatedness reveals that relatedness enhances students’ sense of psychological safety, fostering the development of ICC. These findings align with studies by Ruan and Medwell (2020) and Teng and Bui (2020), highlighting psychological resilience as a critical psychological resource for enhancing ICC.

Competence, whether directly or indirectly through social media interaction and psychological resilience, demonstrates the strongest effect on ICC. This is likely because competence is directly linked to students’ learning and adaptive abilities, significantly boosting their confidence and performance in intercultural interactions. Its importance is particularly pronounced in language learning and cultural adaptation.

## Research discussion

### Academic contributions

This study, grounded in Self-Determination Theory (SDT), systematically explored the influence mechanisms of autonomy, competence, and relatedness on the Intercultural Communicative Competence (ICC) of international students learning Chinese in China, incorporating social media interaction and psychological resilience as mediating variables. The main academic contributions of this research are as follows:

Expanding the application scope of SDT in cross-cultural contexts This study applied SDT to the field of cross-cultural education, quantifying the direct and indirect effects of autonomy, competence, and relatedness on ICC. By validating the critical role of these three core psychological needs in enhancing students’ ICC, the research enriches the applicability of SDT in multicultural contexts and addresses empirical gaps in SDT’s use in cross-cultural education and adaptation studies.

#### Revealing the mediating role of social media interaction and psychological resilience

This study highlights the important mediating roles of social media interaction and psychological resilience in the development of Intercultural Communicative Competence (ICC). Firstly, social media interaction was shown to be a critical platform for students to adapt to local cultures and facilitate language learning and cultural exchange. The findings demonstrate that autonomy, competence, and relatedness can indirectly enhance students’ ICC by increasing social media interaction, providing empirical support for the application of social media in cross-cultural education. Secondly, psychological resilience was identified as a core mediating variable influencing ICC, showcasing its key role in helping students manage cultural stress, regulate emotions, and foster intercultural understanding. The combined effect of psychological resilience and competence is particularly significant in improving students’ ICC, offering a new perspective on the value of psychological traits in cross-cultural adaptation research. These findings further enrich the theoretical foundation of cross-cultural education and emphasize the crucial role of mediating variables in enhancing ICC.

#### Providing a comparison of influence strengths and validation of the theoretical model

This study compared the influence strengths of autonomy, competence, and relatedness on ICC, revealing that competence had the most significant impact, followed by autonomy, with relatedness showing a relatively weaker effect. Additionally, the research validated the influence pathways involving two mediating variables through structural equation modeling (SEM), presenting a more precise mechanism for the development of Intercultural Communicative Competence. This provides robust theoretical support and an analytical framework for related studies.

### Practical recommendations

Based on the findings of this study, the following practical recommendations are proposed to assist educational institutions, teachers, and policymakers in effectively supporting international students learning Chinese in China and enhancing their Intercultural Communicative Competence (ICC).

#### Enhance students’ competence

Provide high-quality Chinese language courses and cultural training to help students improve their language skills and cultural knowledge, thereby boosting their sense of competence. Develop personalized learning plans and offer tailored guidance based on students’ language proficiency and learning needs, allowing them to gain successful learning experiences and build confidence.

#### Utilize social media as an educational tool

Encourage students to use social media platforms such as WeChat and Weibo for language practice and cultural exchange, fostering their intercultural interactions. Teach students how to use social media safely and effectively for learning and communication, helping them avoid cultural misunderstandings and communication barriers.

#### Foster students’ psychological resilience

Provide psychological counseling services and stress management workshops to help students cope with academic and life pressures, thereby enhancing their resilience. Develop specialized resilience training programs to teach students strategies for handling challenges and difficulties, improving their adaptability.

### Research limitations and future directions

Although this study verified the mechanisms by which autonomy, competence, and relatedness influence Intercultural Communicative Competence (ICC) through social media interaction and psychological resilience, several limitations remain. First, as data were collected via voluntary online surveys, there may be a self-selection bias, where students who are more motivated or confident in intercultural contexts are more likely to participate. Second, the sample focused only on international students studying Chinese in China, which may limit the generalizability of the results. Third, while participants came from various nationalities, the study did not control for specific cultural background differences, which may influence how students interpret psychological needs or communication behaviors. Fourth, the use of a cross-sectional design makes it impossible to reveal long-term dynamic relationships. Additionally, other potential factors influencing ICC, such as cultural intelligence and personality traits, were not thoroughly considered. Future research can extend to students from different cultural backgrounds and regions, adopt longitudinal designs to explore causal mechanisms, introduce more variables such as cultural intelligence and digital literacy, and compare the effects of different social media platforms to construct a more comprehensive theoretical framework and validate the effectiveness of interventions.

## Data Availability

The original contributions presented in the study are included in the article/supplementary material, further inquiries can be directed to the corresponding author.
